# Barrier Height Prediction by Machine Learning Correction of Semiempirical Calculations

**DOI:** 10.1021/acs.jpca.2c08340

**Published:** 2023-03-06

**Authors:** Xabier García-Andrade, Pablo García Tahoces, Jesús Pérez-Ríos, Emilio Martínez Núñez

**Affiliations:** † AWS Networking Science, Dublin D04 HH21, Ireland; ‡ Department of Electronics and Computer Science, University of Santiago de Compostela, Santiago de Compostela 15782, Spain; § Department of Physics, Stony Brook University, Stony Brook, New York 11794, United States; ∥ Institute for Advanced Computational Science, Stony Brook University, Stony Brook, New York 11794-3800, United States; ⊥ Department of Physical Chemistry, University of Santiago de Compostela, Santiago de Compostela 15782, Spain

## Abstract

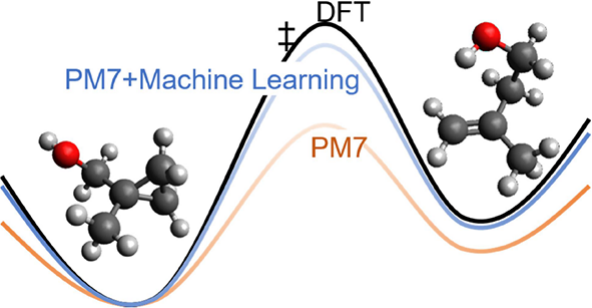

Different machine learning (ML) models are proposed in the present work to predict density functional theory-quality barrier heights (BHs) from semiempirical quantum mechanical (SQM) calculations. The ML models include a multitask deep neural network, gradient-boosted trees by means of the XGBoost interface, and Gaussian process regression. The obtained mean absolute errors are similar to those of previous models considering the same number of data points. The ML corrections proposed in this paper could be useful for rapid screening of the large reaction networks that appear in combustion chemistry or in astrochemistry. Finally, our results show that 70% of the features with the highest impact on model output are bespoke predictors. This custom-made set of predictors could be employed by future Δ-ML models to improve the quantitative prediction of other reaction properties.

## 1 Introduction

Transition state theory (TST) provides a useful means to study the kinetics of elementary chemical reactions.^1^ Depending on the specific version, TST requires a more or less exhaustive knowledge of the potential energy surface of the system.^2^ In the absence of strong tunneling effects, the value of the Gibbs energy of activation Δ*G*^‡^ [Gibbs energy difference between the transition state (TS) and the reactant(s)] is sufficient to predict the rate of reaction. At 0 K, Δ*G*^‡^ is just the electronic energy difference between the TS and reactant including their zero-point vibrational energies (ZPEs), called the barrier height (BH). Although the BH does not include the thermal correction to enthalpy and the entropic contribution, sometimes it is employed as a proxy for the true Gibbs energy of activation. Nevertheless, predicting highly accurate BHs (of sub-kcal/mol accuracy) requires the use of expensive ab initio methods, such as the gold standard coupled cluster including single and double excitations with perturbative triple excitations [CCSD(T)].^3^ Fortunately, today’s state-of-the-art density functionals predict BHs that are rather close to the accurate CCSD(T),^4^ thus being the method of choice for modeling large systems. However, even density functional theory (DFT) becomes prohibitive for biochemical systems or for complex reaction networks of medium-size systems.

With the surge of large computational and experimental data sets, machine learning (ML) is shifting the paradigm to data-driven predictive modeling. This approach has been pursued to predict activation energies and BHs in previous studies.^5−15^ By way of example, Choi et al. developed different ML models to predict activation energies of gas-phase reactions, with the tree-boosting method showing the best performance.^5^ More recently, Green and co-workers have demonstrated that it is possible to predict accurate BHs using a deep learning (DL) model given only reactant and product graphs.^6,8^ Green’s DL model was trained on a gas-phase organic chemistry (GPOC) data set of 12,000 chemical reactions involving carbon, hydrogen, nitrogen, and oxygen. The calculations were carried out at the DFT ωB97X-D3/def2-TZVP quantum chemistry level, which has been shown to predict BHs with a mean absolute error (MAE) of 3.5 kcal/mol against a CCSD(T)-F12 reference.^16^ An updated version of the GPOC is available,^16^ with BHs calculated at the CCSD(T)-F12 level of theory; in a follow-up work our models will be improved using the newest data set. Green and co-workers recently improved their model using fewer parameters and proper data splits to estimate performance on unseen reactions.^8^ In addition, Habershon and co-workers employed this basis set to predict rates of chemical reactions.^17^ Alexandrova and co-workers have also shown that topological descriptors of the quantum mechanical charge density in the reactant state can be used to predict BHs for Diels–Alder reactions.^9^ Hybrid models combining traditional TS modeling and ML are also employed to predict BHs for nucleophilic aromatic substitution reactions in solution.^10^

Semiempirical quantum mechanical (SQM) methods are significantly faster than DFT and provide results with sufficient accuracy when applied to molecules of the same type as those of the training set.^18^ However, except when the interest is in a specific reaction,^19−23^ training sets do not usually include data of TSs, which results in inaccurate BH predictions. In an attempt to model the reactivity of organic reactions with useful accuracy, Stewart developed the SQM method called PM7-TS.^18^ Using a training set of 97 BHs obtained from collections of high-level calculations, the MAE using PM7-TS was 3.8 kcal/mol, as compared with the MAEs for PM7 of 11.0 kcal/mol and for PM6 of 12.2 kcal/mol.^18^ However, Jensen and co-workers benchmarked PM7-TS using BHs for five model enzymes and found an MAE of 19 kcal/mol, while the MAEs for PM6 and PM7 were around 12–15 kcal/mol.^24^ Iron and Janes^25^ have also shown that SQM methods perform very poorly in predicting transition metal BHs: using a new data set with high-accurate energies, the MAEs of PM6, PM7, and PM7-TS are 21.6, 106.4, and 68.2 kcal/mol, respectively. In his PM7 paper, Stewart already acknowledged that the predictive power of PM7-TS was unknown at the time and suggested parameter re-optimization as more BHs became available.^18^

An alternative to parameter optimization is to develop analytical^26^ or ML corrections of the SQM calculations. The latter are usually termed Δ-ML because the model predicts the difference between the benchmark and the approximate baseline calculation (SQM in this case).^27^ There are some examples in the literature of the successful use of ML to improve the accuracy of both DFT^28−30^ and SQM calculations.^31,32^

In this work, we leverage ML to predict BHs with DFT accuracy at the cost of SQM calculations. The model employs multitask deep neural network (DNN), gradient-boosted trees by means of the XGBoost (XGB) interface,^33^ and Gaussian process (GP) regression trained on a curated version of the GPOC data set.^7^ Gradient boosting regression has been successfully applied to predict BHs in Diels–Alder reactions,^9^ and the reactivity of transition metal complexes.^15^ Similarly, GP has shown a great performance in complex potential energy surface fittings,^34−37^ predicting spectroscopic constants of diatomic molecules^38,39^ and second virial coefficients of organic and inorganic compounds.^40^ The selected SQM model was PM7,^18^ which is overall the most accurate method implemented in MOPAC2016.^41^ To fully exploit the SQM calculations, several input features are constructed from the electronic and structural properties of reactant, TSs, and products. Moreover, the model makes different predictions for cases where two TSs exist for the same rearrangement.^42^ A similar synergistic SQM/ML approach to predict activation energies for a diverse class of C–C bond-forming nitro-Michael additions has been recently proposed.^43^

The ML correction proposed in this paper could be employed in conjunction with SQM-based methods for automated reaction mechanism prediction like AutoMeKin.^44−47^

## 2 Methods

### 2.1 Performance of PM7-TS on the GPOC Data Set

**Figure 1:**
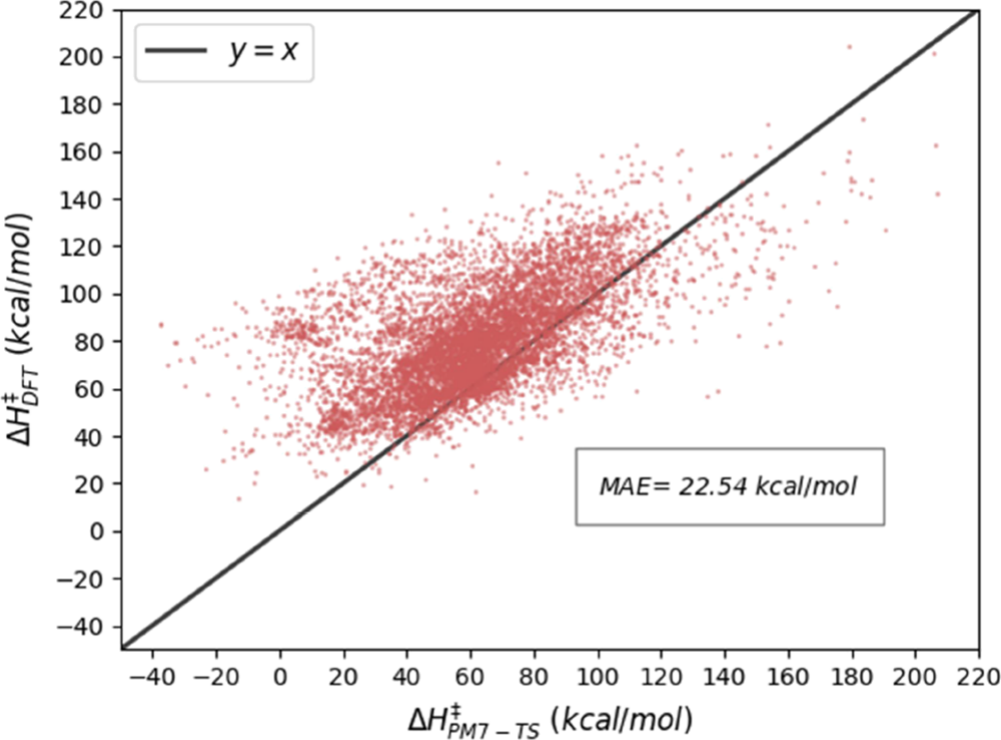
Performance of PM7-TS on the GPOC data set.

Since the accuracy of PM7-TS is uncertain (*vide supra*), its performance was evaluated on the GPOC data set of BHs.^7^Figure 1 shows the correlation between the ωB97X-D3/def2-TZVP BHs and the values predicted by PM7-TS. In general, PM7-TS significantly underestimates the BHs with an MAE of 22.5 kcal/mol, which is in line with the deviation obtained by Jensen and co-workers on a data set of model enzymes^24^ and much greater than the reported error of 3.8 kcal/mol on the training set employed to optimize the PM7-TS parameters.^18^ These results call for an alternative method to predict accurate SQM-based BHs. The proposal of the present work is to employ ML models to correct the SQM values.

### 2.2 Data Set Curation

**Figure 2:**
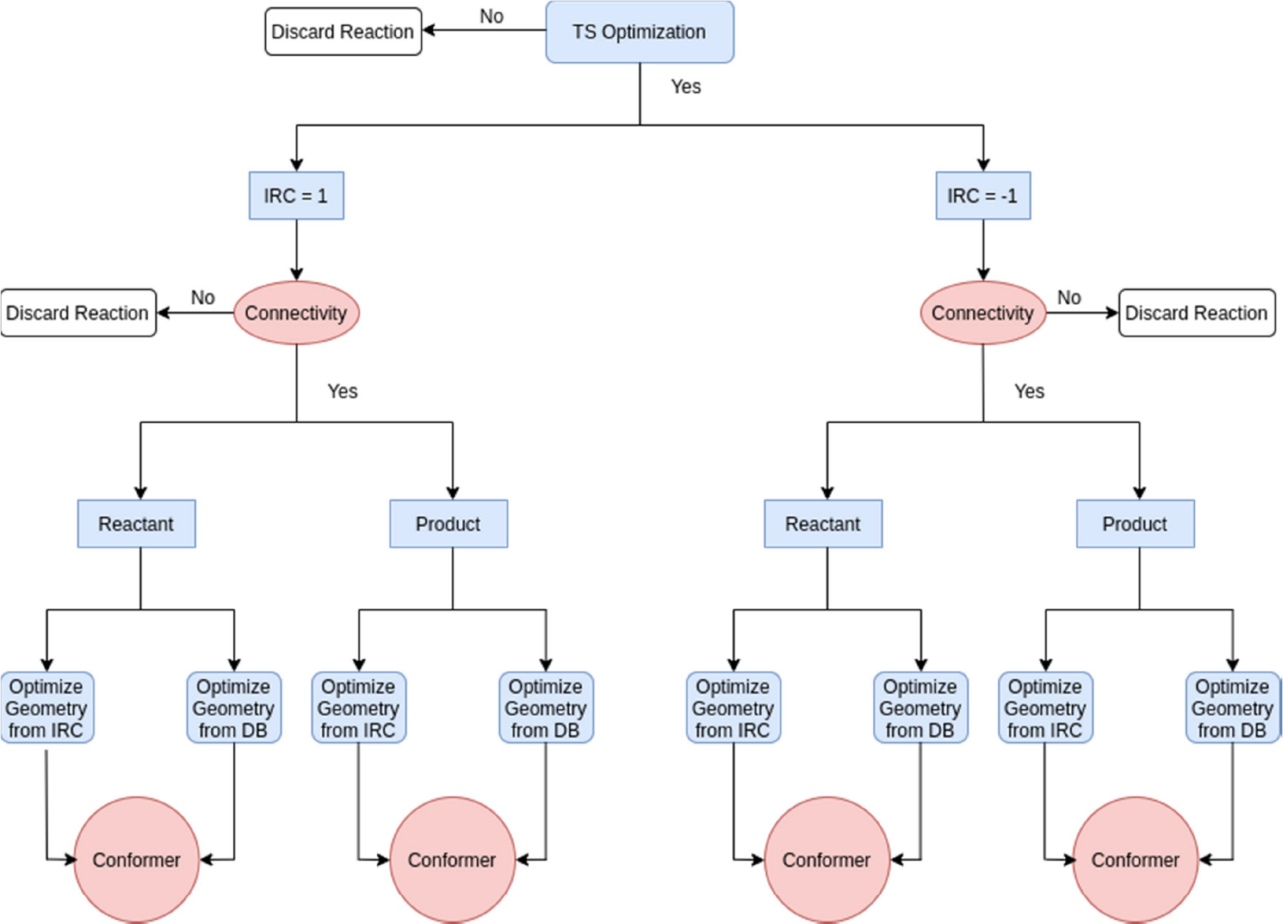
SQM data set generation flow diagram.

The target for our ML models is the difference BH^DFT^ – BH^PM7^, where BH^DFT^ and BH^PM7^ are the BHs obtained at the benchmark (DFT) and PM7 levels, respectively. The GPOC data set developed by Green and co-workers is employed here.^7^ It contains 11,960 reactions, with energies for reactant, TSs, and the product obtained at the ωB97X-D3/def2-TZVP level of DFT. The DFT BHs were directly obtained by subtracting the reactant energy from the TS energy including their ZPEs. Obtaining BHs at the PM7 SQM level entails a more involved process, as several sanity checks are required. All SQM calculations were carried out with MOPAC2016^41^ and the settings employed in the different PM7 calculations are detailed in the Supporting Information (SI). A flow chart diagram explaining how the PM7 BHs were obtained is shown in Figure 2. The first step, labeled as TS optimization in the figure, consists of optimizing the TSs at the PM7 level using as initial guesses the geometries optimized at the DFT level. Some structures could not be optimized at the PM7 level and were discarded. Then, for each successfully optimized TS structure, an IRC calculation^48^ is carried out in each direction (IRC = 1 and IRC = −1 in the figure). The IRC end points are compared with the reactant and product present in the data set. For such a comparison, the eigenvalues of the corresponding adjacency matrices (with their diagonals representing the atomic numbers) were employed.^49^ Obtaining identical eigenvalues ensures that the connectivity of each structure (reactant and product) is the same at both levels of theory (DFT and SQM). When the connectivity differs for either reactant or product, the reaction is discarded. Otherwise, both the IRC end point and the structure from the data set are optimized at the PM7 level and compared to ensure they present the same conformation. For this last comparison that involves 3D structures, the eigenvalues of a weighted adjacency matrix are employed.^49^

From the initial 11,860 reactions, 8355 survived this screening process, meaning that roughly 70% of the samples could be utilized in our model. This means that our approach is limited to situations where a TS can be optimized. The use of reverse BHs did not lead to a major improvement during the training of the model but increased the computational cost, so this form of data augmentation was discarded.

An exploratory data analysis (EDA) of the curated GPOC data set was then carried out. The detailed results of our EDA are collected in the SI. Reactions in the data set contain up to seven heavy atoms (C, N, or O) per molecule and consist of unimolecular reactions leading to one or more products (although most reactions are isomerizations).

### 2.3 Machine Learning Models

**Figure 3:**
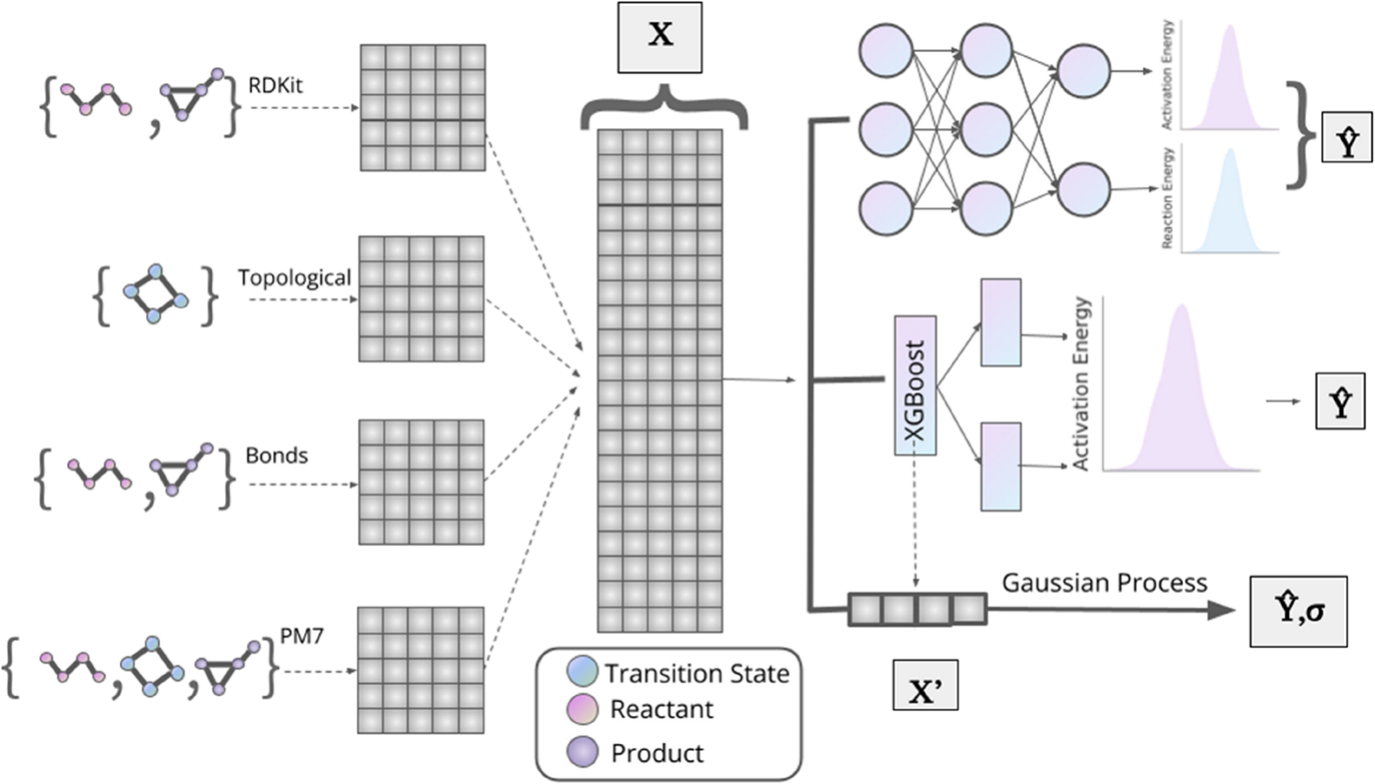
Workflow for the prediction of barrier heights using three machine learning models to correct SQM barrier heights. Two types of descriptors are employed: standard RDKit-based and our own custom set that comprises three subtypes. These features ***X*** are input to DNN and XGB regressors, whereas the input features for the GP are labeled by ***X′***, which is a subset of ***X*** informed by the feature importance results from the XGB model. The DNN model predicts the BH and the energy difference between the reactant and product. The XGB and GP models predict the BH only.

Figure 3 shows the workflow for the three ML models employed in this study to correct SQM BHs. A crucial step of the models is the calculation of a set of descriptors (or input features) that encode the most useful information present in every reaction. Our models employ two types of descriptors: (a) standard RDKit-based descriptors and (b) a custom set based on the SQM calculations and chemical intuition. Figure 3 also shows how every species in the reaction (namely, TS, reactant, or product) contributes to each set of descriptors.

The first set of descriptors *X*_RDKit_ is obtained from the cheminformatics library RDKit.^50^

Each descriptor of this type *X*_RDKit, *i*_ is calculated as follows:
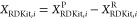
1where *X*_RDKit, *i*_^R^ and *X*_RDKit, *i*_^P^ refer to the *i*th RDKit descriptor of the reactant and product, respectively. If a specific descriptor remains invariant in the reaction (like the molecular weight), the raw value is employed instead of eq 1. The *X*_RDKit_ set contains 132 descriptors (see the SI for details).

Besides the above standard set of descriptors, a custom set is also employed in this work. This set is specifically tailored to extract the most relevant features of chemical reactions. It comprises information on the topology of the TS, the number of bonds that change in the reaction, and results from the PM7 calculations.

An advantage of our model is that the approximate TS structures calculated at the PM7 level of theory can be employed as input features. Specifically, the 3D geometries are converted into molecular graphs, represented in the form of adjacency matrices, using the definitions employed in AutoMeKin.^47^ From the molecular graphs, some topological descriptors *X*_topol_ can be constructed. These include Randic’s connectivity index,^51^ the spectral gap (or lowest nonzero eigenvalue of the Laplacian matrix, λ_1_^TS^), the Estrada index,^52^ or the Zagreb index;^53^ the full list of topological descriptors can be found in the SI. The Laplacian matrix defined as *D* – *A* (with *D* and *A* being the degree and adjacency matrices, respectively) is calculated from a weighted adjacency matrix to account for 3D structures of the TSs.^47^ Topological descriptors provide a measure of the extent of branching or the tightness of the TS structure.

The subset *X*_bonds_ includes the number of broken and formed bonds of each type, i.e., all pairings of H, C, N, and O atoms. For instance, this set includes the descriptors +CO and −CH, which refer to the number of formed CO bonds and a number of broken CH bonds, respectively.

The last subset of descriptors *X*_PM7_ capitalizes on the PM7 calculations. This set includes the BH, a rough proxy for the rate constant *e*^–BH^, the imaginary frequency at the TS ν_1_^TS^, and differences between ZPEs of reactant, product, and TS: ZPE^R^, ZPE^P^, ZPE^TS^, respectively. The subset also comprises electronic descriptors like the eigenvalues of the bond order matrix calculated at the TS, the global “hardness”,^54^ and Mulliken’s electronegativity^55^ at the TS (η^TS^ and α^TS^, respectively), and differences between the self-polarizability^56^ of reactant π_R_^s^ and product π_P_^s^. While some of these descriptors are readily available from a frequency calculation at the TS, others are obtained using the keyword SUPER in MOPAC.

Having defined the input features, a correlation matrix was built where each entry represents the Pearson coefficient *r* for every pair of descriptors. A threshold was established such that if the correlation coefficient exceeds this value, one of the descriptors is dropped from the input features. The threshold was optimized by cross-validation and set to *r* = 0.9.

These stacked descriptors are input to the three models depicted in Figure 3: DNN, XGB, and GP. DNN works in a multitask approach, where the output includes, besides the BH, the energy difference between the reactant and product. This approach has been shown to enhance predictions and generalization power, even if our interest is only in the BHs.^6,57^ The architecture of the DNN model (number of hidden layers and number of neurons) as well as other hyperparameters were fine-tuned in a fivefold cross-validation fashion using a grid search, considering some hyperparameters to be orthogonal.

Nevertheless, since our set of descriptors consists of heterogeneous tabular data and the amount of data is limited by DL standards, we decided to use two alternative approaches, that perform better in this case, XGB and GP.^58^ In particular, we chose XGB^33^ implementation of gradient boosting techniques, which achieves state-of-the-art results and provides sparsity-aware algorithms particularly suited for our data set. In this case, hyperparameters were optimized by means of the Bayesian optimization library Optuna,^59^ using fivefold cross-validation as well. Furthermore, considering that gradient boosting techniques that rely on decision trees as weak learners assign higher importance to descriptors that will be more relevant for other models, we find a more succinct descriptor *X*’ containing only 49 features to feed in the GP model. The GP model, after being exposed to the training data, generates a multivariate Gaussian prior distribution that by means of Bayesian inference leads to a posterior distribution for the test set. Thus, leading to a prediction with a confidence interval based on the inherent Bayesian nature of the model.

Following common practices in the ML literature as well as considering the size of the data set, the data was split into 85% training, 5% validation, and 10% testing. The first data set partitioning was made prior to any hyperparameter optimization phase, relying on random splits. For the validation set, we relied on cross-validation.

TensorFlow,^60^ XGB,^33^ and MATLAB^61^ were used for the DNN, XGB, and GP models, respectively. The specifics of the models can be looked up in the provided repository or notebook.

## 3 Results and Discussion

### 3.1 Performance of the Machine Learning Models

**Figure 4:**
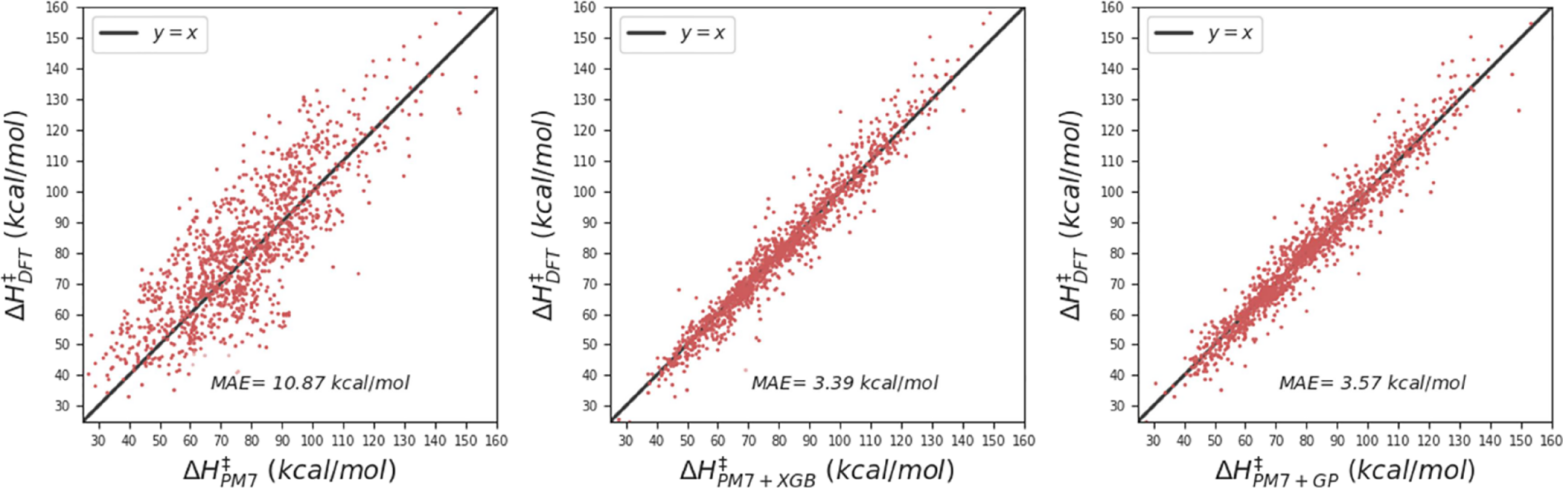
Barrier height predictions at DFT, PM7, PM7 + XGB, and PM7 + GP levels.

The MAEs obtained in this work using ML models DNN, XGB, and GP are 3.69, 3.39, and 3.57 kcal/mol, respectively. Figure 4 shows the correlation between the reference (DFT) vs the predicted values of the BHs obtained with the XGB and GP models for the test set. In the interest of simplicity, the figures only display results for our best models (XGB and GP).

**Figure 5:**
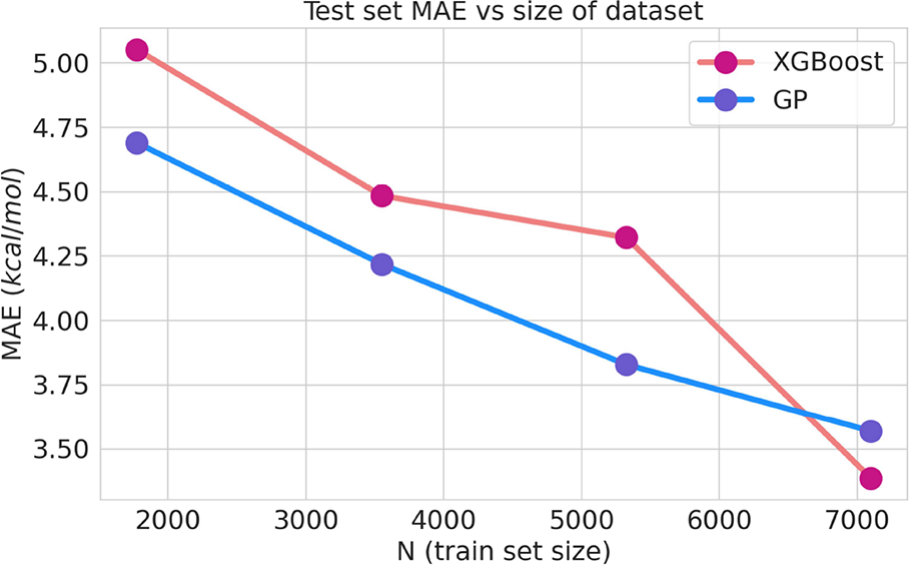
MAEs vs train set size for the GP and XGBoost models.

The performance of our models is comparable to Green’s for the same number of training points^6^ and markedly better than either PM7 or PM7-TS. The MAEs vs the number of training data points for our models are shown in Figure 5, where MAEs decrease as new training data points are included. In comparing both models, GP is more data efficient, as it performs better for a smaller number of data points. Nevertheless, GP seems to converge as new training data points are considered, which is not the case for XGB. The latter outperforms GP when the total data set is used becoming the preferable model for larger data sets. For 7100–7500 data points, the MAEs obtained in this work are 3.57 and 3.39 kcal/mol for GP and XGB, respectively, which can be compared with a value greater than 3.6 kcal/mol obtained by Green and co-workers.^6^ It should be noted here that the procedure employed in this work cannot exploit the sort of data augmentation employed in Green’s work by including reverse reactions because many of our features refer to the TSs structures, which are common to both direct and reverse reactions.

**Figure 6:**
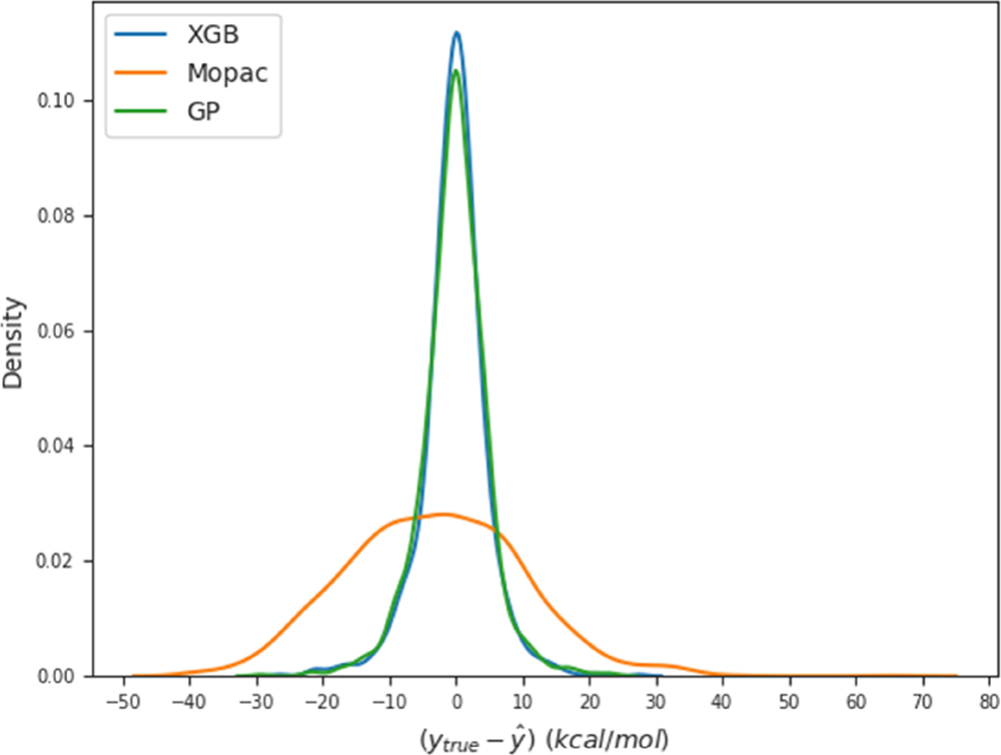
Error distribution on the target variable for the ML models (XGB and GP) in comparison with the one obtained directly from the MOPAC calculations.

Figure 6 shows the error distribution on the target variable for XGB and GP in comparison with the results obtained with MOPAC’s PM7 calculations. For the XGB and GP models, most reactions show an error smaller than 10 kcal/mol, in stark contrast with PM7-MOPAC predictions, thus showing a superior accuracy of the ML models with respect to PM7. As mentioned in the Introduction, a new data set is available,^16^ with BHs calculated at the CCSD(T)-F12 level of theory. The performance of our models against a CCSD(T) reference could be worse than the one obtained here. The most recent and accurate data set^16^ will be employed in a separate study to improve on our current models.

### 3.2 Interpretability

Models can be interpreted in terms of their feature importances, i.e., how much a certain feature contributes to the prediction. Feature importances are obtained in present work from the SHAP values,^62^ which resort to game-theoretic approaches to measure the contribution to the model output by each descriptor. The underlying principle is to measure the expected change in output when using different combinations of descriptors.

**Figure 7:**
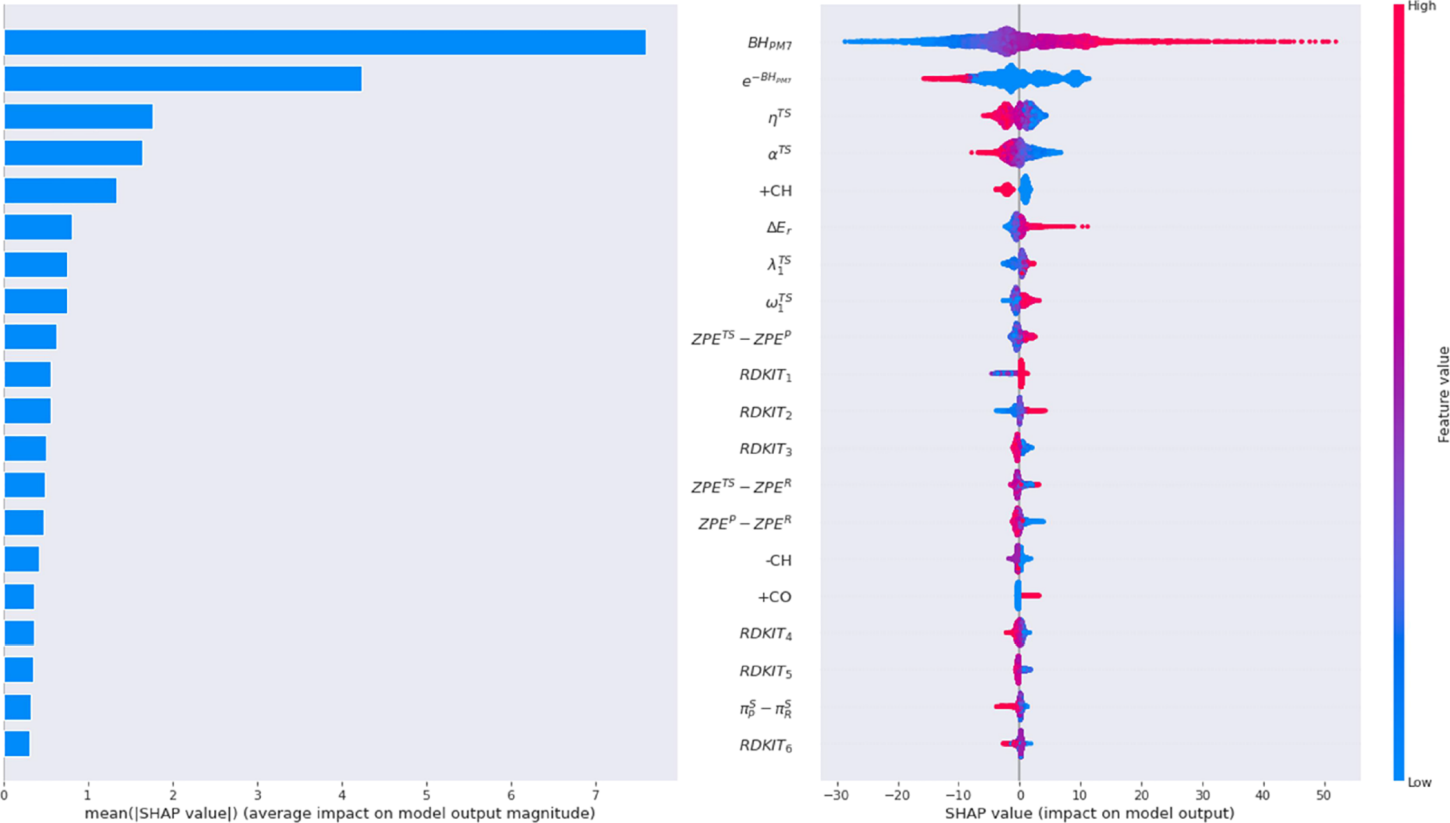
SHAP values for the top 20 most relevant descriptors and their impact on model output.

Figure 7 shows a SHAP (SHapley Additive exPlanations) summary plot, which displays the magnitude and direction of a feature’s effect. Interestingly, 70% (14/20) of the most important features belong to the custom set. As expected, the features with the greatest impact on the model output are the values of the PM7 barrier height BH_PM7_ and the proxy for the rate constant *e*^–BH_PM7_^. The MAEs obtained with XGB without BH_PM7_ and *e*^–BH_PM7_^ are 4.10 and 3.47 kcal/mol, respectively. While they encode the same information, and gradient boosting (or any other model relying on decision trees as weak learners) are, in principle, invariant with respect to monotonic transformations, in this case, we included both the transformed and original descriptor. This can lead to collinearity, but XGB can handle these situations, and based on both feature importance and the increase in model performance, we decided to keep both descriptors.

Figure 7 also shows that PM7 tends to underestimate high BHs and vice versa, which is reflected by the positive impact on the model output for high BHs. Our result is in agreement with a recent ML model to predict activation energies from DFT calculations, where the DFT-computed activation energy was also the most important feature.^10^

The “hardness” η^TS^ and Mulliken’s electronegativity α^TS^ calculated at the TS also rank very high on the global feature importance plot. Using Koopman’s theorem,^63^ they can be approximated as η = (ε_LUMO_ – ε_HOMO_)/2 and α = – (ε_LUMO_ + ε_HOMO_)/2, where ε_LUMO_ and ε_HOMO_ are the energies of the lowest unoccupied molecular orbital (LUMO) and of the highest occupied molecular orbital (HOMO), respectively. These descriptors have been employed as an index to predict the chemical behavior and reactivity^64−70^ and even to locate TSs.^71^ The value of η decreases as the molecule departs from its equilibrium position, attaining a minimum at the TS. The LUMO/HOMO energies have also been employed to predict activation energies in Diels–Alder reactions.^11^

With similar impacts on the model output, the absolute value of the imaginary frequency ω_1_^TS^ and the lowest nonzero eigenvalue of the Laplacian λ_1_^TS^ (or spectral gap) at the TS are also among the most important descriptors according to Figure 7. Both provide a measure for the tightness of the TS structure, with the imaginary frequency also containing information on the mass of the atoms involved in the reaction coordinate.

The number of formed CH and CO bonds (+CH and +CO, respectively), the number of broken CH bonds (−CH), ZPE differences among reactant, TS, and product, the PM7 reaction enthalpy (Δ*E*_r_), or the self-polarizabilities (π_R_^s^ and π_P_^s^) also contribute among the most important features. The importance of the number of formed/broken bonds of different types in the model output can be explained by the accuracy of SQM methods predicting bond energies, which strongly depends on the bond type.^72^

RDKit descriptors considered important include SMR_VSA (RDKIT 1), LabuteASA (RDKIT 2),^73^ and VSA_EState2 (RDKIT 7). These descriptors grant a measure of the approximate accessible van der Waals surface area per atom. Other relevant descriptors are as follows: Balaban J (RDKIT 3), referring to the connectivity distance of the molecular graph,^74^ Chi0_v^75^ (RDKIT 6), which is also a topological-based descriptor, and MolLogP^76^ (RDKIT 5), which refers to atom-based partition coefficients.

**Figure 8:**
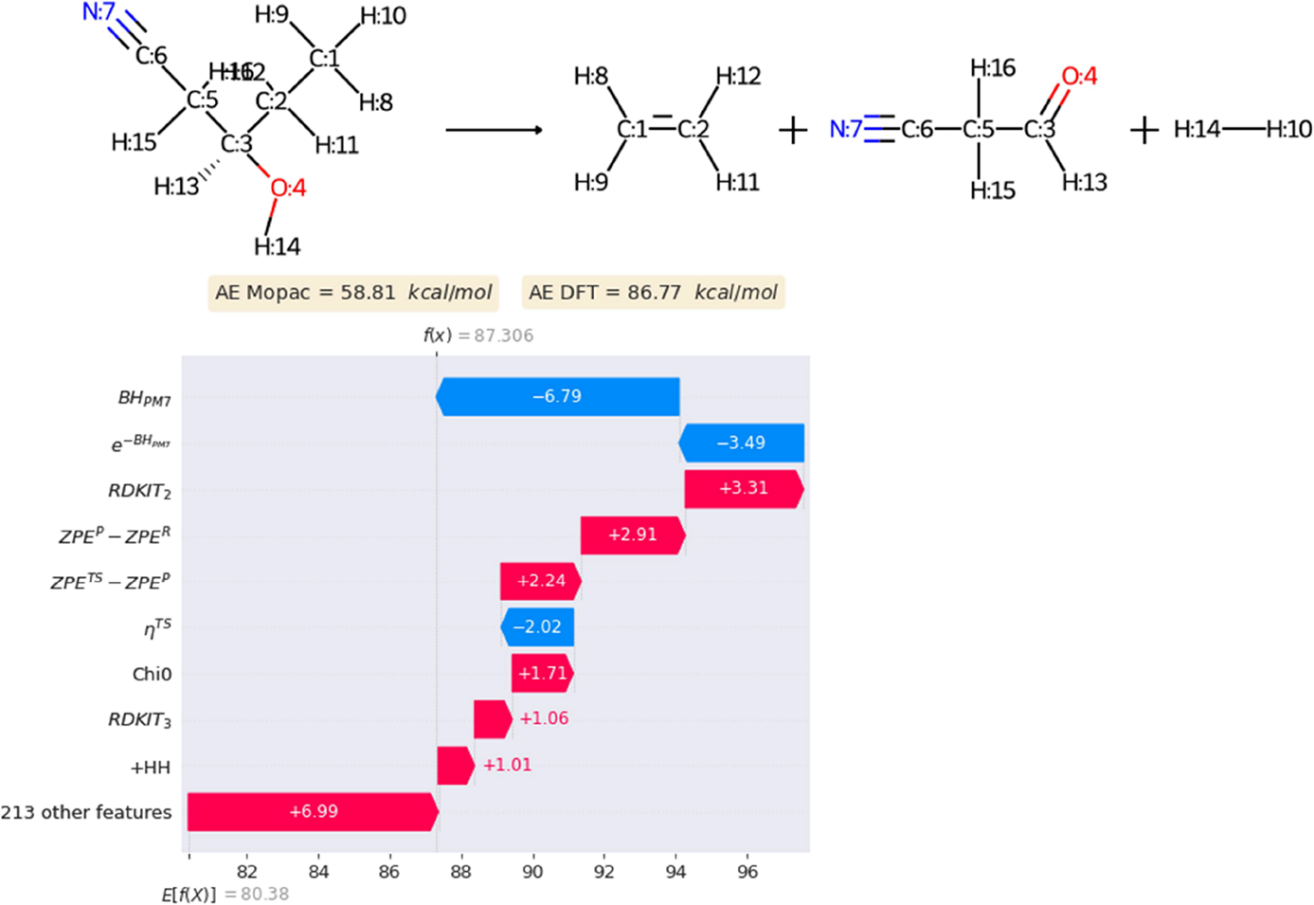
Model output interpretation for a single reaction.

Figure 8 showcases how SHAP values can be used for interpretation of a single reaction. It shows the descriptors that contribute the most to shift the prediction of the model from its average (expected) prediction. Not surprisingly, BH_PM7_ and *e*^–BH_PM7_^as well as other descriptors of Figure 7 contribute significantly also for this particular reaction. Additionally, since this reaction involves the formation of molecular hydrogen, the number of formed H–H bonds (+HH) is also an important descriptor.

### 3.3 Entropic Effects

In mechanistic and kinetics studies of chemical reactions the quantity of interest is the Gibbs energy of activation Δ*G*^‡^, rather than the BH. The reason is that the former includes enthalpic and entropic corrections to the electronic and ZPE energies. Reaction channels that are not very competitive at low temperatures/energies might become predominant at high temperatures/energies because of entropic factors.^77^ Therefore, the prediction of Δ*G*^‡^ is crucial when the interest is the kinetics and the determination of the predominant mechanism.

The calculation of Δ*G*^‡^ is straightforward when the geometries and vibrational frequencies of the reactant and TS are available. The values of Δ*G*^‡^ have been obtained in this work at different temperatures using the thermochemistry module of AutoMeKin^47^ for the reference and SQM calculations using the rigid rotor/harmonic oscillator approximation. In the absence of a scaling factor for the ωB97X-D3/def2-TZVP vibrational frequencies, the value of 0.9914 was employed; this is the recommended value for the related ωB97X-D/def2-TZVP model chemistry.^78^ Furthermore, the PM7 vibrational frequencies were corrected using the recommended scaling factors.^79^

**Figure 9:**
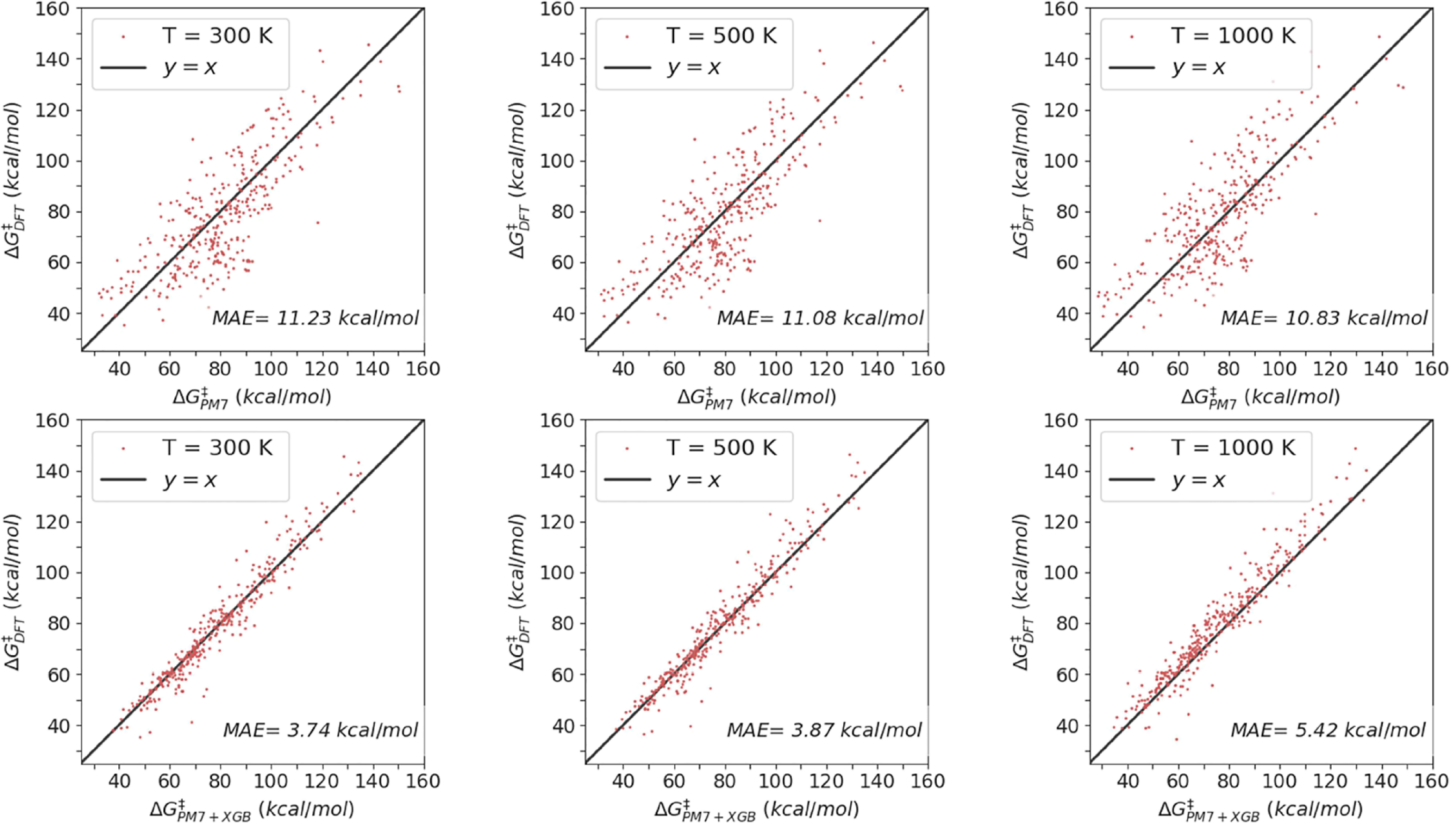
Correlation of the DFT, PM7, and PM7 + XGB values for the Gibbs energy difference between the reactant and transition state Δ*G*^‡^ at *T* = 300, 500, and 1000 K.

Figures 9 and S11 display the correlation between the reference (DFT), the PM7, PM7 + XGB, and PM7 + GP predictions for Δ*G*^‡^ at three different temperatures: 300, 500, and 1000 K, respectively. At the two lowest temperatures, Δ*G*^‡^ predictions are roughly of the same accuracy as those for the BH. However, for the highest temperature of 1000 K, the ML predictions start to deteriorate and the MAE at this temperature is 5.30 kcal/mol for the GP model. A clear improvement to the model would be to use a multitask ML model to correct the SQM vibrational frequencies. Nevertheless, the current accuracy of our models significantly improves the PM7 accuracy, and it may suffice for fast screening of reaction networks.

## 4 Conclusions

The main conclusions of this work are summarized below:

a) Cheap SQM calculations can be leveraged to obtain DFT-quality BHs by means of ML.
b) The MAEs of our ML models (multitask DNN, gradient-boosted trees by means of the XGB interface, and GP regression) are of the same magnitude as those obtained in previous work.
c) The analysis of the models shows that the custom-made descriptors obtained from the MOPAC calculations are, in general, considered more important than those obtained from standard cheminformatics libraries.
d) Our MOPAC-based descriptors could be widely adopted in future quantitative predictions of reaction properties.
e) Our ML models could be used for screening large reaction networks, or they could be implemented in automated reaction mechanism programs based on SQM calculations.

## Supporting Information Available

Exploratory data analysis; details of the hyperparameter optimization; descriptor explanation; links to the data and code employed in this work; and free energies of activation obtained with the GP model (PDF)
