# Supplementary material for: Barrier Height Prediction by Machine Learning Correction of Semiempirical Calculations
Source: J Phys Chem A. 2023 Mar 6;127(10):2274–83. doi: 10.1021/acs.jpca.2c08340 (PMC10845151; doi:10.1021/acs.jpca.2c08340)
Supplement: Supplementary file 1 — jp2c08340_si_001.pdf [file jp2c08340_si_001.pdf]

Supporting Information for:

Barrier Height Prediction by Machine Learning Correction of Semiempirical Calculations

Xabier García-Andrade,[a,1] Pablo García Tahoces[b] , Jesús Pérez-Ríos[c,d] and Emilio Martínez Núñez*[e]

[a]AWS Networking Science.
[b]Department of Electronics and Computer Science, University of Santiago de Compostela 15782, Spain
[c]Department of Physics, Stony Brook University, Stony Brook, New York 11794, USA
[d]Institute for Advanced Computational Science, Stony Brook University, Stony Brook, NY 11794-3800, USA
[e]Department of Physical Chemistry, University of Santiago de Compostela 15782, Spain. Email address: emilio.nunez@usc.es

[1] Work done prior to joining AWS

Contents

1. Exploratory Data Analysis: S3
2. Hyperparameter Optimization: S7
3. Descriptor explanation:.. S9
4. Reproducibility:... S10
5. Free energies of activation: S11
6. References: S12

1. Exploratory Data Analysis:

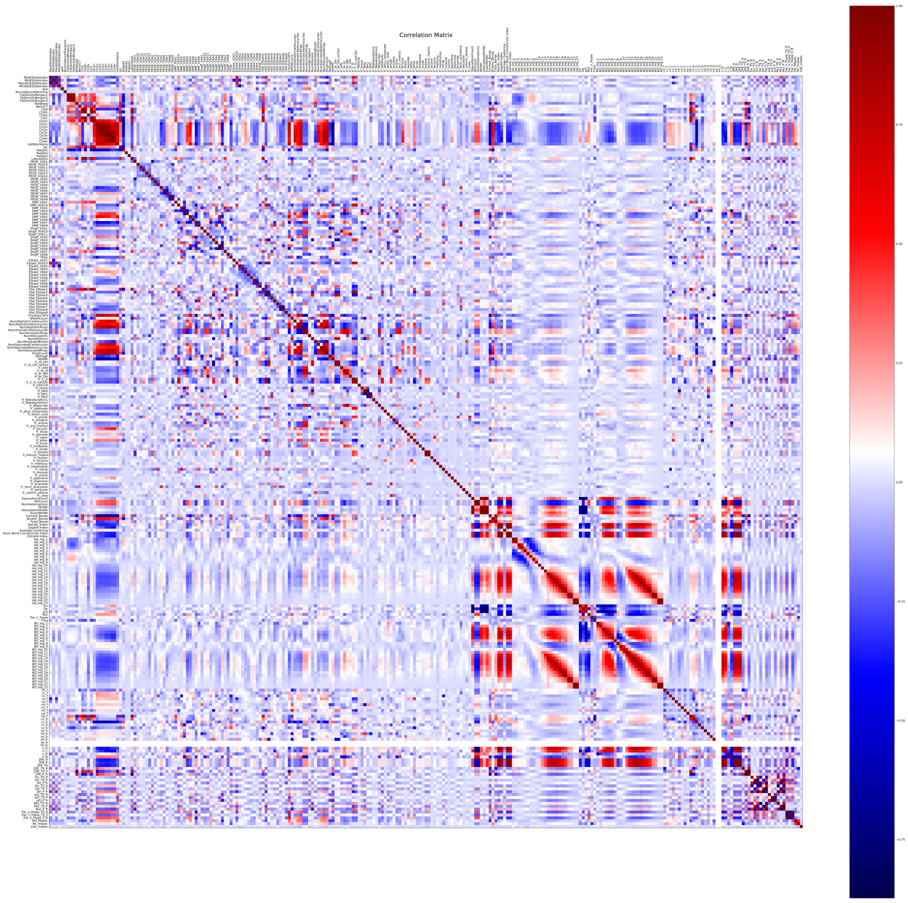

Figure S1. Correlation matrix, computing the pairwise Pearson coefficient for each pair of descriptors.

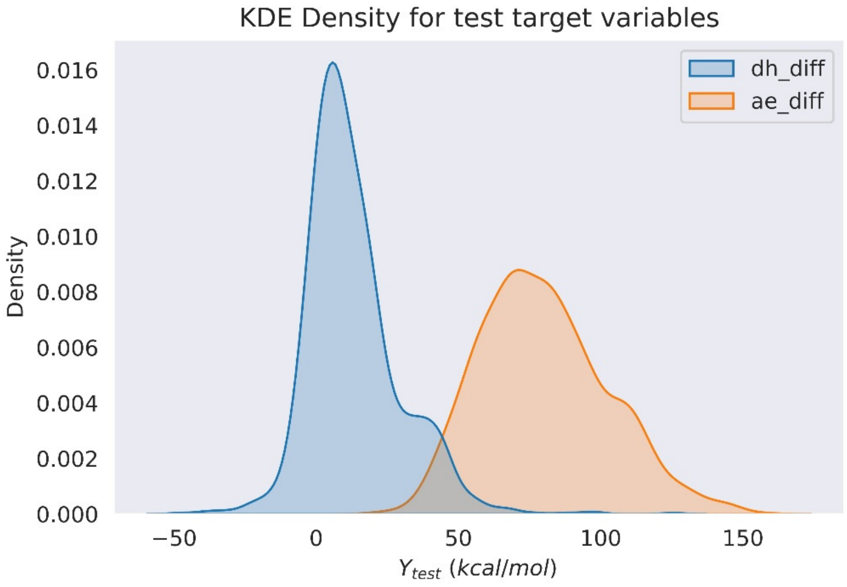

Figure S2. Test set target variables distribution.

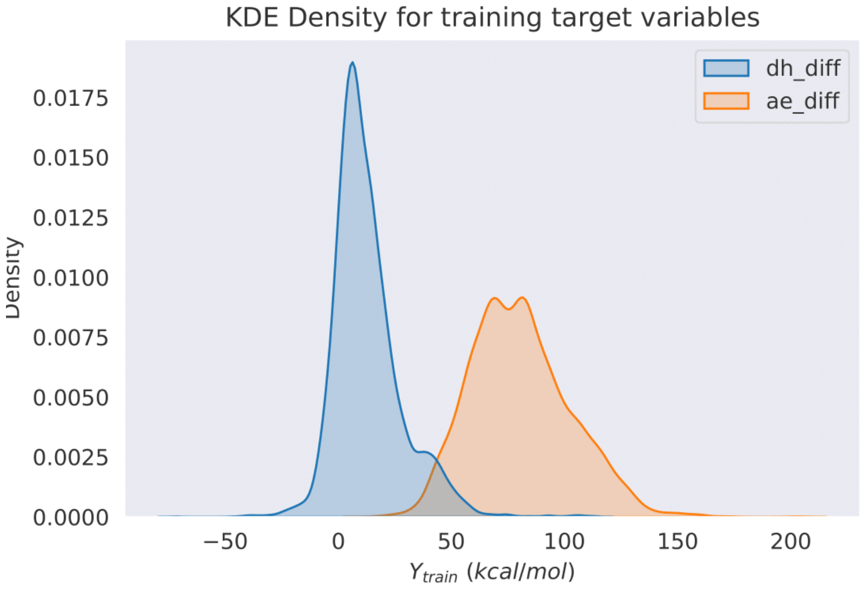

Figure S3. Train set target variables distribution.

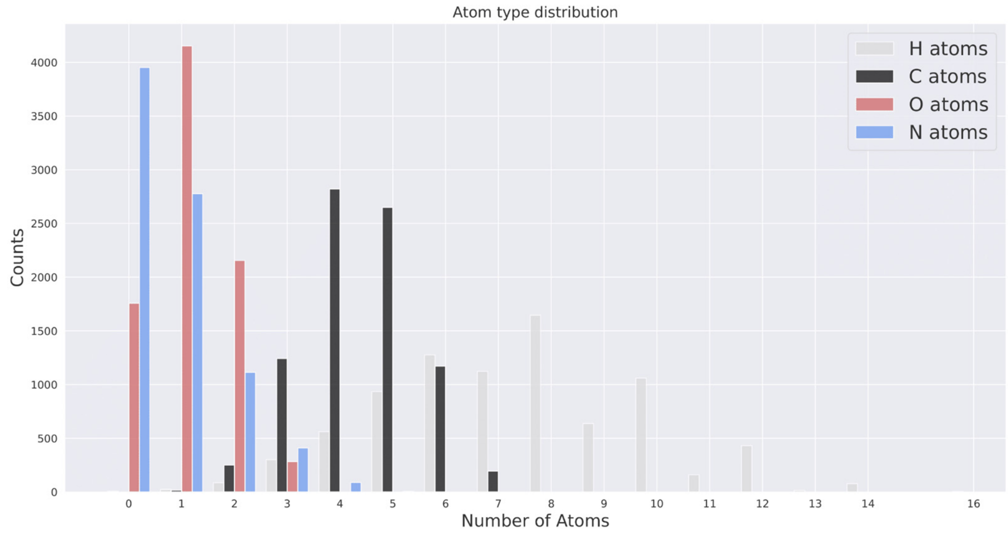

Figure S4. Type of atom count per reaction in the whole database.

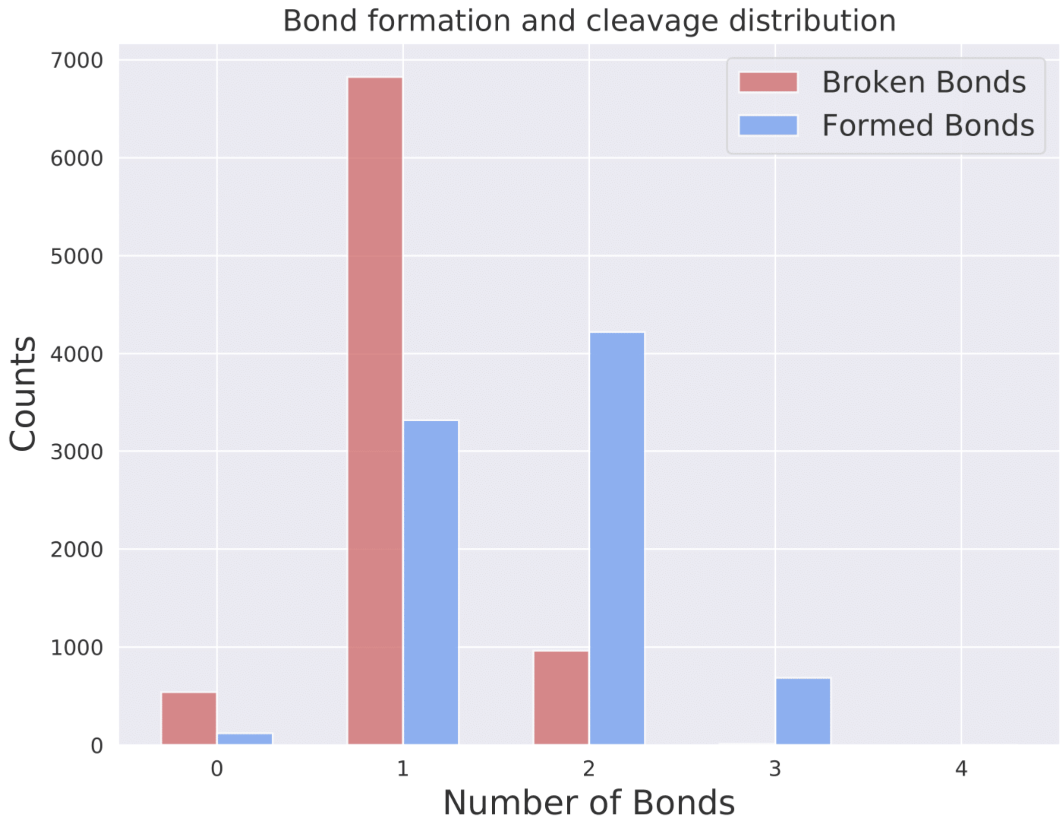

Figure S5. Frequency of the number of broken and formed bonds per reaction.

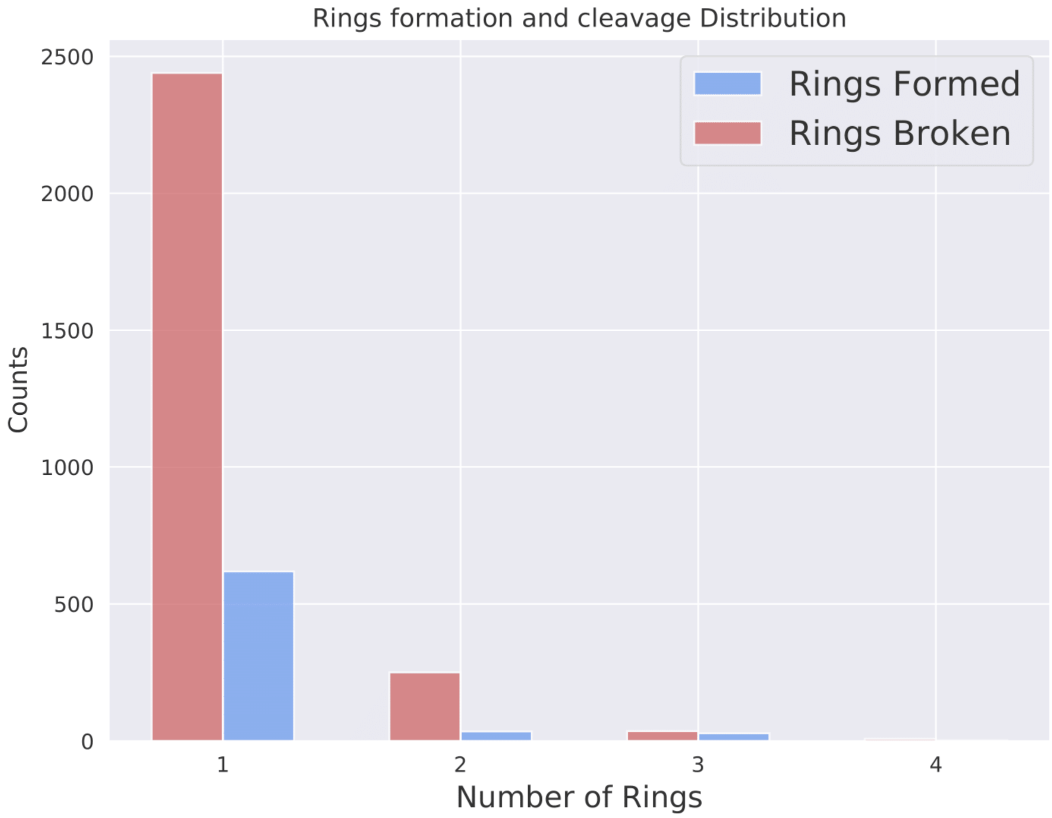

Figure S6. Frequency of ring formation and ring cleavage per reaction.

2. Hyperparameter Optimization:

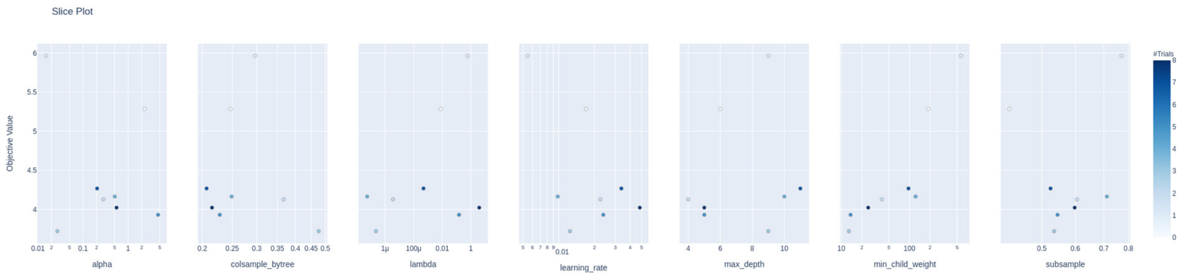

Figure S7. Optuna values per trial in the hyperparameter optimization iterations.

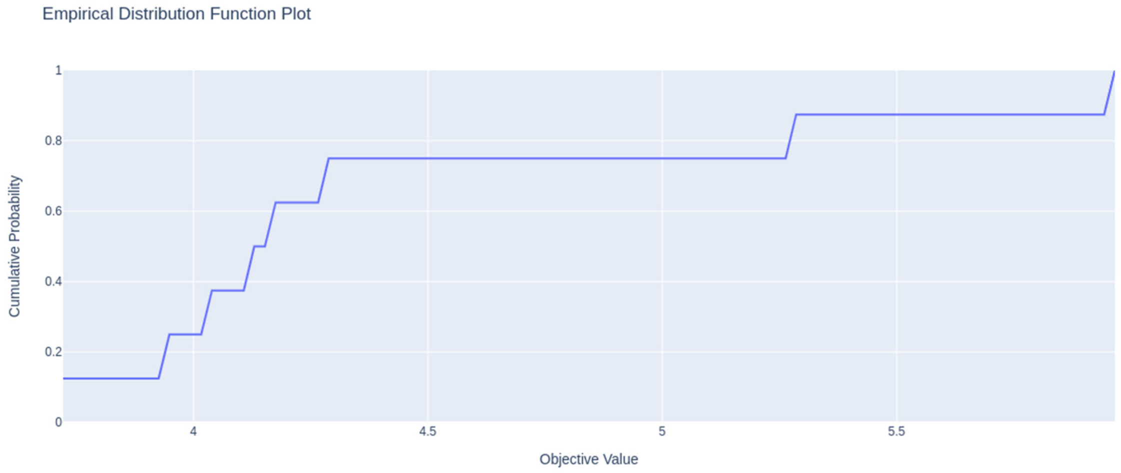

Figure S8. Optuna empirical cumulative probability.

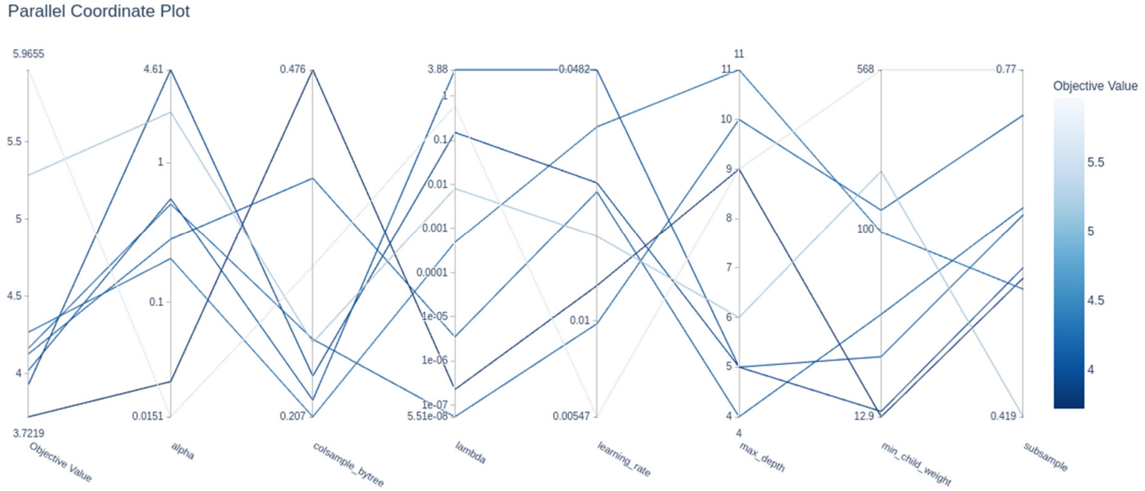

Figure S9. Interactions between the hyperparameters and objective value .

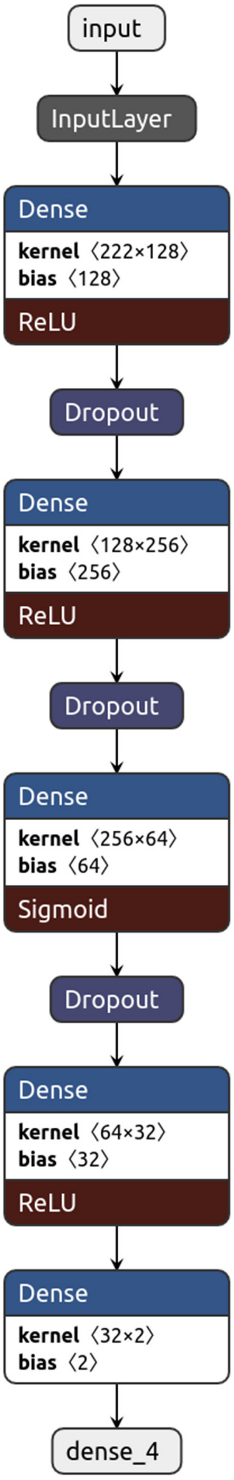

Figure S10. DNN architecture after hyperparameter tuning.

3. Descriptor explanation:

List of descriptors in the dataset (using the same name convention as in the provided dataset):

- Graph-theoretic (topological) descriptors:
    - randic_index: captures the contributions per bond to each vertex. [1]
    - zagreb_index: graph invariant descriptor [2].
    - avg_clustering: descriptor that measures how close an atom is to its neighbors. [3]
    - atom_bond_conn_idx: provides an estimate to the strain energy of cycloalkanes. [4]
    - estrada_index:
    - lap_eig: Eigenvalues of the transition state Laplacian matrix, which need to be padded to the maximum number of atoms in the dataset.
- MOPAC descriptors:
    - Dn, De, piS, Mul, Par_n_Pople: Nucleophilic and Electrophilic delocalizabilites, Self-Polarizability, Mulliken Electronegativity, and Parr & Pople absolute hardness. These are quantities derived from the density matrix [5].
    - Freq: first real-valued frequency of a transition state.
    - BO_eigs: Eigenvalues of the bond order matrix provided by the MOPAC calculation.
    - ZPE: Zero Point Energy computed using MOPAC.
    - Differences of the previous quantities between the entities involved in the reaction (e.g. ZPE_TS_R refers to subtracting the ZPE of the reactant from the ZPE of the transition state.
    - AE_mopac and exp_mopac: barrier height and minus the exponential of the barrier height computed at MOPAC level of theory.
    - DH_mopac: reaction enthalpy computed at MOPAC level of theory.
- Bond descriptors:
    - Broken_bonds: Number of broken bonds.
    - Formed_bonds: Number of formed bonds.
    - Total_bonds: Number of total bonds.
    - Counts of the type of atoms in the reaction (e.g. n_H is the count of Hydrogen atoms in the reaction).
    - Counts of the type of bonds involved in the reaction (e.g. co_f refers to the number of C-O bonds formed in the reaction).
- RDKit descriptors [6].

Initially, every descriptor provided by the RDKit library was considered, and later a fraction was dropped because of the preprocessing stage (based on correlation). In order to look up the specific set of descriptors used as input after preprocessing, as well as the input descriptors used to train the GP model, the reader can check Section 4 of this SI on reproducibility.

4. Reproducibility:

The data and code are available in the following repository:
https://github.com/XabierGA/SemiDFT

The interested reader can reproduce the whole ML workflow by downloading the notebooks on the repository or run it on the browser at:
https://colab.research.google.com/drive/1nj_dEx5j6g1O2Bk4KGvKxBq0Q7DuYMJ1?usp=sharing

Keywords used for MOPAC calculations:

TS optimization
```
ts precise cycles=5000 t=500 ddmax=0.2 denout threads=1
```

IRC calculation
```
irc= 1 precise cycles=5000 t=500 oldens threads=1
```

The different keywords can be looked up here: http://openmopac.net/manual/allkeys.html

5. Free energies of activation:

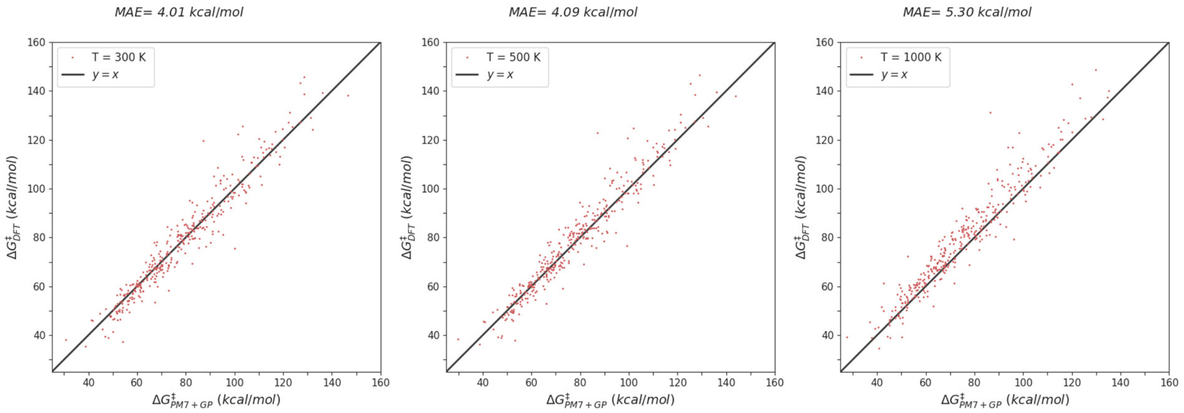

Figure S11. Correlation of the DFT, and PM7+GP values for the Gibbs energy difference between the reactant and transition state $\Delta G^{\ddagger}$ at T = 300, 500 and 1000 K.

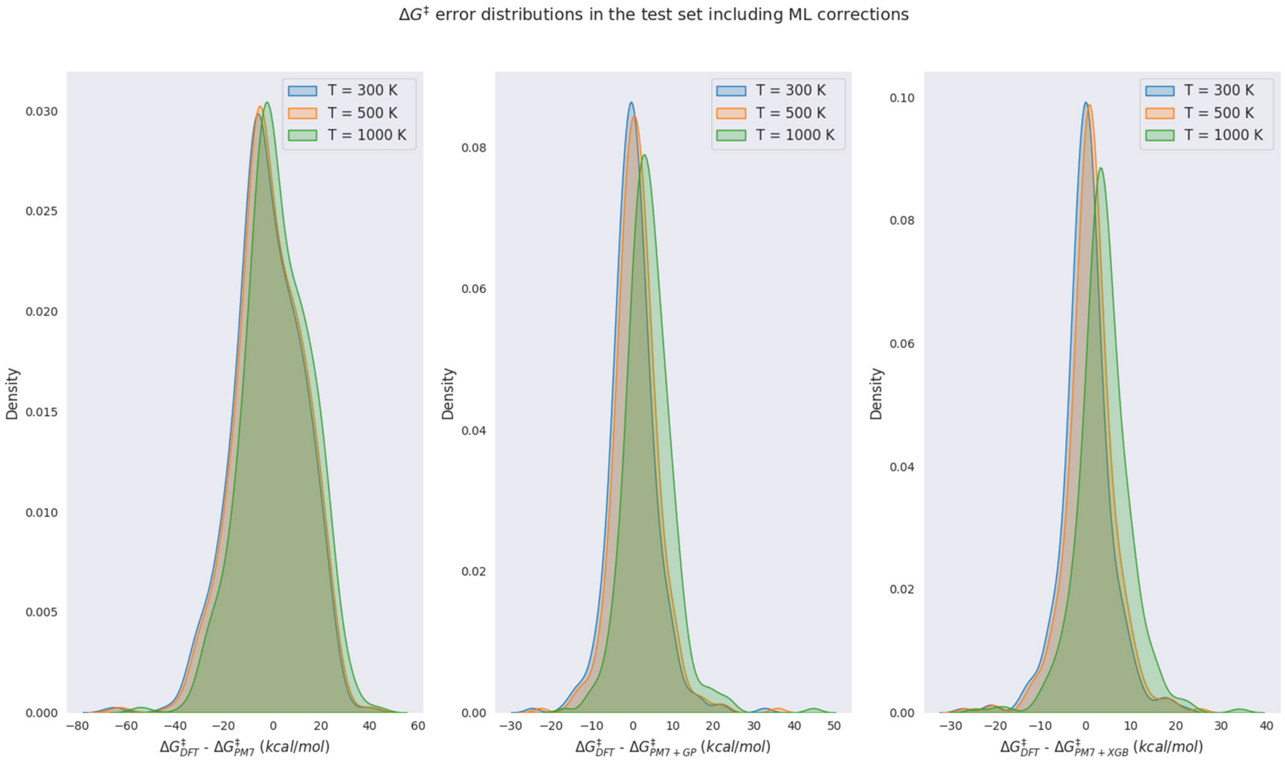

Figure S12. $\Delta G^{\ddagger}$ error distributions in the test set including ML corrections at T = 300, 500 and 1000 K.

6. References:

1. M. Randic, J. Am. Chem. Soc., 1975, **97**, 6609–6615.
2. K. Xu, Applied Mathematics Letters, 2011, **24**, 1026–1030.
3. D. J. Watts and S. H. Strogatz, Nature, 1998, **393**, 440–442.
4. K. Ch. Das, Discrete Applied Mathematics, 2010, **158**, 1181–1188.
5. J. J. P. Stewart, Stewart Computational Chemistry, Colorado Springs, CO, USA, http://openmopac.net/manual/super.html
6. G. Landrum, RDKit: Open-source cheminformatics (2016). https://www.rdkit.org.
